# Invasive alien plants are phylogenetically distinct from other alien species across spatial and taxonomic scales in China

**DOI:** 10.3389/fpls.2023.1075344

**Published:** 2023-09-07

**Authors:** Achyut Kumar Banerjee, Fengxiao Tan, Hui Feng, Xinru Liang, Jiakai Wang, Minghui Yin, Hao Peng, Yuting Lin, Nannan Zhang, Yelin Huang

**Affiliations:** ^1^State Key Laboratory of Biocontrol and Guangdong Provincial Key Laboratory of Plant Resources, School of Life Sciences, Sun Yat-sen University, Guangzhou, Guangdong, China; ^2^College of Natural Resources and Environment, South China Agricultural University, Guangzhou, Guangdong, China; ^3^Chinese Academy of Sciences Key Laboratory of Mountain Ecological Restoration and Bioresource Utilization and Ecological Restoration Biodiversity Conservation Key Laboratory of Sichuan Province, Chengdu Institute of Biology, Chengdu, Sichuan, China

**Keywords:** community assembly, invasion process, phylogenetic structure, invasional meltdown, phylogenetic niche conservatism, biological invasion, phylogeny

## Abstract

**Introduction:**

Phylogenetic relatedness is one of the important factors in the community assembly process. Here, we aimed to understand the large-scale phylogenetic relationship between alien plant species at different stages of the invasion process and how these relationships change in response to the environmental filtering process at multiple spatial scales and different phylogenetic extents.

**Methods:**

We identified the alien species in three invasion stages, namely invasive, naturalized, and introduced, in China. The occurrence records of the species were used to quantify two abundance-based phylogenetic metrics [the net relatedness index (NRI) and the nearest taxon index (NTI)] from a highly resolved phylogenetic tree. The metrics were compared between the three categories of alien species. Generalized linear models were used to test the effect of climate on the phylogenetic pattern. All analyses were conducted at four spatial scales and for three major angiosperm families.

**Results:**

We observed significantly higher NRI and NTI values at finer spatial scales, indicating the formation of more clustered assemblages of phylogenetically closely related species in response to the environmental filtering process. Positive NTI values for the invasive and naturalized aliens suggested that the presence of a close relative in the community may help the successful naturalization and invasion of the introduced alien species. In the two-dimensional phylogenetic space, the invasive species communities significantly differed from the naturalized and introduced species, indicating that established alien species need to be phylogenetically different to become invasive. Positive phylogenetic measures for the invasive aliens across the spatial scales suggested that the presence of invasive aliens could facilitate the establishment of other invasive species. Phylogenetic relatedness was more influenced by temperature than precipitation, especially at a finer spatial scale. With decreased temperature, the invasive species showed a more clustered assemblage, indicating conservatism of their phylogenetic niche. The phylogenetic pattern was different at the family level, although there was a consistent tendency across families to form more clustered assemblages.

**Discussion:**

Overall, our study showed that the community assemblage became more clustered with the progression of the invasion process. The phylogenetic measures varied at spatial and taxonomic scales, thereby highlighting the importance of assessing phylogenetic patterns at different gradients of the community assembly process.

## 1 Introduction

Biological invasions are considered one of the most important global problems experienced by ecosystems (Simberloff et al., 2013). Phylogenetic relatedness of the alien species to the existing community members is hypothesized to facilitate [(pre-adaptation hypothesis; (Ricciardi and Mottiar, 2006)] or hinder [(Darwin’s naturalization hypothesis; (Daehler, 2001)] successful establishment of alien species in the community. The alternative views of these two hypotheses (also called Darwin’s naturalization conundrum) are often resolved by considering the gradients of community assembly (Thuiller et al., 2010). Biotic interactions in a similar environment at a small spatial scale and environmental heterogeneity at a large scale influence the phylogenetic relationship between the community members (Procheş et al., 2008). Numerous studies have investigated the phylogenetic relationship between native and alien species to provide evidence supporting these two hypotheses [e.g., (Procheş et al., 2015; Divisek et al., 2018; Omer et al., 2022)].

However, the introduction of an alien species may also occur in a community with existing alien species belonging to different stages of the invasion process, i.e., introduced, naturalized, and invasive. Biotic interactions (e.g., mutualism) between the alien species at a small spatial scale and environmental factors (e.g., changes in habitat conditions) at a large spatial scale may influence the community assembly process (Simberloff and Von Holle, 1999). The majority of studies on the transition of alien species along the invasion process have been conducted at large spatial scales, often at the country level considering whole flora (e.g., Australia (Gallagher et al., 2015), or for a family [e.g., Proteaceae in South Africa (Moodley et al., 2013)]. These studies either considered phylogenetic uniformity between species of different invasion stages (Gallagher et al., 2015) or tested phylogenetic signals in the factors that could influence the transition (Maurel et al., 2016). Therefore, knowledge of the phylogenetic relationship between the alien species of different invasion stages is rare. Understanding the environmental influence on large-scale phylogenetic relationships across the invasion process may provide important insights into the community assembly process.

This study addressed this knowledge gap by characterizing phylogenetic relationships between alien community members at different spatial and taxonomic scales. Ecological traits are often phylogenetically conserved (Donoghue, 2008), and therefore, species in harsher environments tend to be more closely related (Wiens and Donoghue, 2004). At a large spatial scale, environmental filtering (and not biotic interactions) influences phylogenetic relatedness by sorting phylogenetically closely related alien species in a community (Qian and Sandel, 2017). Therefore, we first hypothesized that the phylogenetic relatedness of alien species should vary across the spatial scale, and the environmental influence will be more pronounced at a large spatial scale, where phylogenetically closely related species will form a more clustered assemblage.

Secondly, among the three stages of the invasion process, the invasive species have been found to possess certain ecophysiological traits (e.g., specific leaf area, height) that aid them in adapting to a range of environmental conditions (Gallagher et al., 2015). Moreover, few studies that have compared functional traits based on phylogenetic history between the native and alien species revealed that the successful invaders need to be functionally different from the native species to occupy novel trait space (Divisek et al., 2018). Adaptations in key traits of the invasive species could arise from standing genetic variation and/or rapid evolution of phenotypically plastic traits during range expansion, particularly in response to ecogeographical variations (Prentis et al., 2008; Hodgins et al., 2018). Although such rapid adaptations are often attributed to their ability to occupy novel niches in the introduced range (Pyron et al., 2015), the fundamental niches of the invasive lineages have been conserved (Liu et al., 2020). Therefore, from an evolutionary perspective, we hypothesized that niche conservatism might increase the phylogenetic clustering of the invasive species compared to that of the aliens that failed to naturalize and naturalized species that failed to invade when environmental filters sort phylogenetically closely related alien species in a community.

Thirdly, the observed phylogenetic pattern can vary depending on the taxonomic scope (whole flora or family-specific) and phylogenetic framework (based on phylogenetic trees or a non-phylogenetic approach) (Qian and Sandel, 2017). As species of the same family are more likely to have similar responses to environmental gradients, we expected that the phylogenetic pattern should be more pronounced at smaller phylogenetic extents. Because individual families have different evolutionary histories, the phylogenetic patterns should also vary among them.

We tested these three hypotheses for the alien plant species present in China. The history of alien plant introduction into China goes back more than 2000 years, and many aliens have become naturalized and subsequently invasive (Axmacher and Sang, 2013). With increased globalization and the gradual opening of China’s economy to the global markets in the 21st century, the novel introduction of alien species is expected to increase. Deciphering the phylogenetic relationship pattern across the invasion continuum in China has received recent attention. Previous studies have provided important insights into the phylogenetic relationships between native and alien species (Qian et al., 2022a), native and naturalized alien species (Qian et al., 2022b), and naturalized and invasive alien species (Qian et al., 2022c). However, phylogenetic relationships between alien species of different invasion stages and the influence of these relationships on the community assembly process at multiple spatial and taxonomic scales are rarely investigated in a single analysis [but see (Zhang et al., 2021)].

Specifically, the objectives of this study were to determine: 1) the variation of phylogenetic relatedness of alien species across spatial scales (hypothesis 1), 2) whether the three groups of alien species (introduced, naturalized, and invasive) show similar phylogenetic patterns (hypothesis 2), and 3) how the phylogenetic relationships change depending on the taxonomic scale (whole flora and family-specific) of investigation (hypothesis 3). We addressed these objectives by relating phylogenetic patterns of alien species across spatial scale, invasion process, and taxonomic scope with climate as an environmental filter.

## 2 Materials and methods

### 2.1 Species selection

We created a comprehensive list of alien plant species in China from three nationwide checklists published until 2021, namely, naturalized alien plants of China [n = 861; (Jiang et al., 2011)], the checklist of the Alien Invasive Plants in China [n = 464; (Ma and Li, 2018)], and the Global Register of Introduced and Invasive Species (GRIIS) - China Version 1.3 [n = 459; (Zhao et al., 2020)]. The species names were standardized using the WorldFlora package (Kindt, 2020) in R version 4.0.2 (R Core Team, 2020). The duplicates, synonyms, infraspecific taxa, and artificially hybridized species were removed. We used the taxize package (Chamberlain and Szöcs, 2013) in R to exclude the non-angiosperm species, as their long branches in a phylogeny, compared to that of angiosperms, could result in unusually high phylogenetic measures (Letcher, 2010). The origin status (native or alien to China) and invasion status (introduced, naturalized, and invasive) of the selected species were ascertained based on the consensus approach of the three national checklists mentioned above and two global databases (details provided in **Supplementary Note 1**). This resulted in a list of 811 alien species, which were categorized as – introduced (i.e., the alien species which are not yet naturalized and invasive, n=193), naturalized (i.e., the alien species which have become naturalized but not yet invasive, n=275), and invasive (i.e., the alien species which have become invasive, n=343).

The occurrence records (latitude and longitude) of species in China were extracted from the Global Biodiversity Information Facility (GBIF.org, 22 February 2022) and digital herbarium sheets, accessed from the database of National Specimen Information Infrastructure (NSII, 30 October 2021) by using the accepted species names. Locality descriptions were used to georeference occurrence data lacking geographic coordinates at a precision level of two decimal degrees. In cases where location information was too vague or in large geographic areas (e.g., national parks), the centroid of the described polygon was used to define the coordinates of the study. All coordinate values were captured in decimal for the latitude and the longitude. The occurrence records were arranged by species names, and the duplicate coordinates for a species were removed. The points falling into the ocean were removed. To avoid the effect of spatial autocorrelation, occurrence records were spatially rarefied within a distance of 5 km (the resolution being consistent with that of the bioclimatic variables used here) by using SDMtoolbox version 2.5 (Brown, 2014) in ArcMap 10.7.1 (ESRI, 2011). After the consolidation of data, a total of 87,660 occurrences (GBIF: 17,924; NSII: 69,736) were obtained for 706 species (introduced = 165, naturalized = 222, invasive = 319) that were included in this study (see **Supplementary Data 1** for the species and occurrence records).

### 2.2 Phylogeny construction

**Figure 1:**
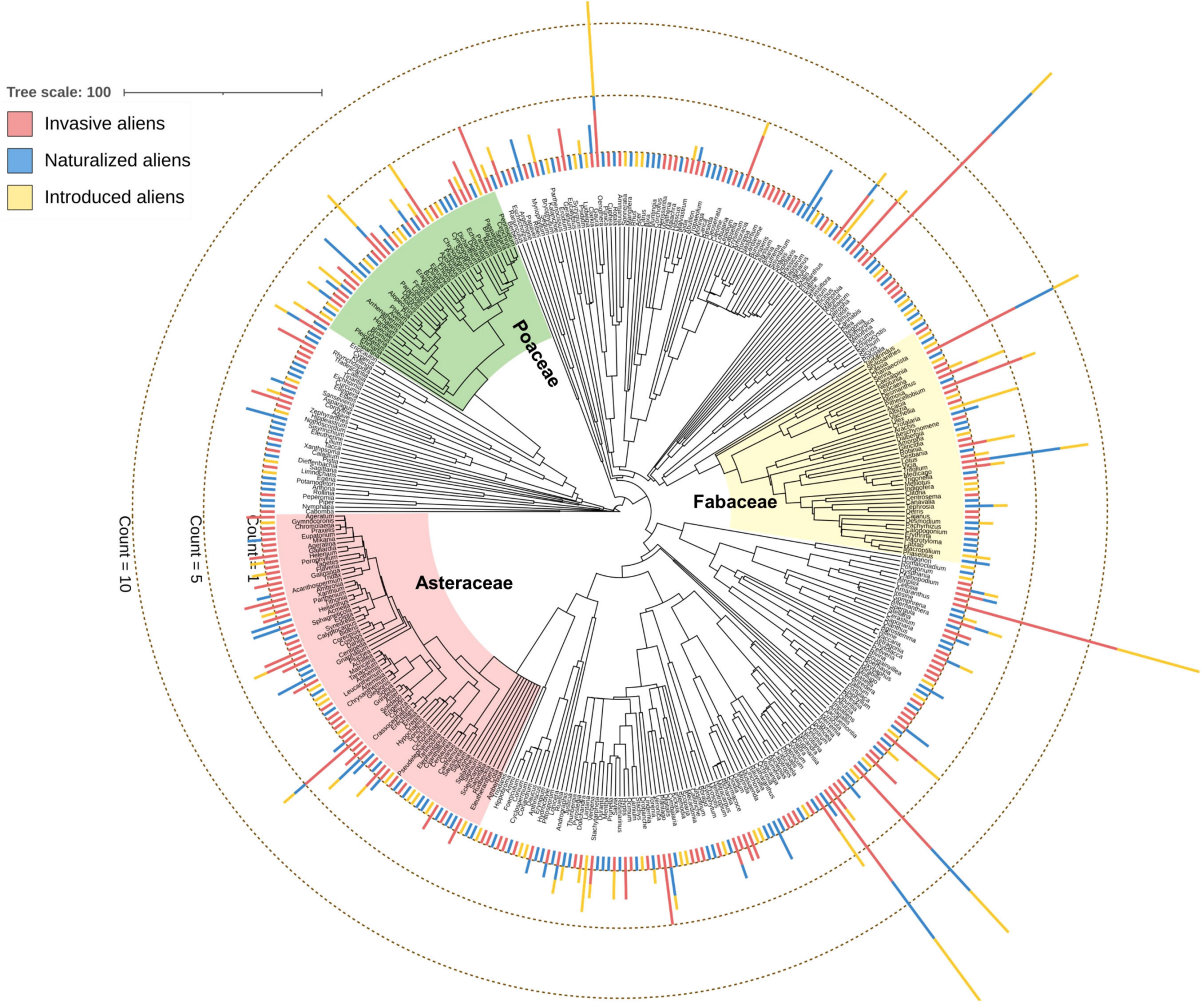
Genus-level phylogenetic tree of the 397 angiosperm genera of 706 alien angiosperm species considered in this study. The stacked bars at the tip of the genus names indicate the number of species of three alien species categories (see **Supplementary Data 1** for the number of species belonging to the three alien species categories). The clades of three major families analyzed separately in this study are indicated.

We used a ‘mega-tree’ approach to generate a highly resolved phylogenetic tree (**Figure 1**) by using the V.PhyloMaker package (Jin and Qian, 2019) in R. This package uses the mega-tree derived primarily from the largest dated phylogeny for seed plants developed by Smith and Brown (2018), based on molecular data from GenBank and data from Open Tree of Life project. We used the function build.nodes.1 to define the basal node of the genus as the most recent common ancestor of all the tips in the largest cluster of the genus. Among the three scenarios used to add genera and species absent from the mega-tree, we used scenario 3, which is particularly suitable for resolving phylogeny at the species level and identifying patterns of phylogenetic properties along environmental gradients (Qian and Jin, 2015). The tree was visualized using the Interactive Tree of Life (iTOL) version 3 (Letunic and Bork, 2016).

### 2.3 Estimation of phylogenetic relatedness

The phylogenetic relatedness was tested from the net relatedness index (NRI) and the nearest taxon index (NTI). These indices were developed by (Webb, 2000) and have been used to quantify the phylogenetic relatedness of species in assemblages in previous studies, including those on alien species [e.g., (Carvallo et al., 2014)]. NRI is a richness-standardized measure of the mean phylogenetic distance (MPD) between all pairs of species in a sample. NTI is the richness-standardized version of the mean nearest taxon distance (MNTD), i.e., the average phylogenetic distance from each species to its closest relative. Both metrics are calculated based on an expected value and variance of MPD or MNTD from a null model. We used Phylocom version 4.2 (Webb et al., 2008) to estimate the NRI and NTI values. Null model 2 of Phylocom was used to shuffle species names across the tips of the phylogenetic tree 9999 times. Positive values of these indices indicate phylogenetic clustering, whereas negative values indicate phylogenetic overdispersion (Webb et al., 2002).

**Figure 2:**
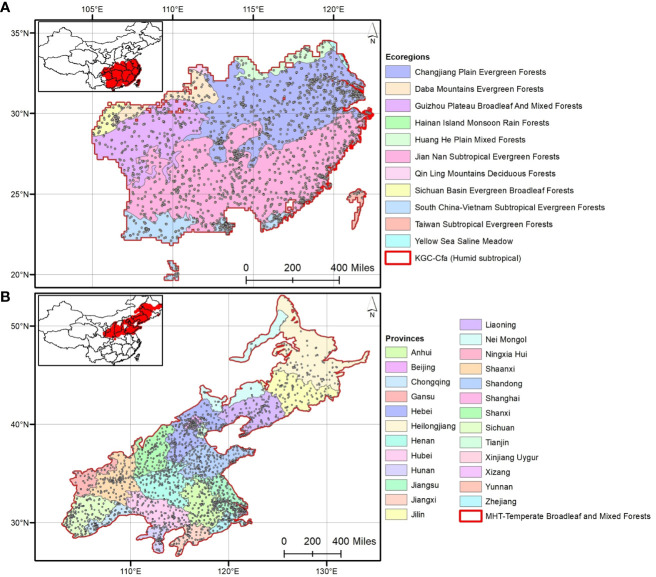
The four spatial scales considered in this study to analyze phylogenetic relationships: **(A)** out of 16 Köppen-Geiger climate classes (KGCs), the humid subtropical KGC is shown that contains 11 out of 47 ecoregions; **(B)** out of seven major habitat types (MHTs), the temperate broadleaf and mixed forests is shown distributing across 25 out of 34 provinces in China. The selected KGC and MHT are marked in red.

Phylogenetic relatedness was measured at four spatial and two taxonomic scales. The spatial scales were determined based on four different tiers of community assembly: major habitat types (MHTs; n=7), Köppen-Geiger climate classes (KGCs; n=16), geographic provinces (PROs; n=34), and ecoregions (ECOs; n=47) (**Figure 2**). The shapefiles of MHTs and ECOs were downloaded from the geospatial database of The Nature Conservancy (https://geospatial.tnc.org/; date of access: 22 February 2022), and the provincial map (level– 1) of the country was downloaded from the GADM version 4.0.4 (https://gadm.org/data.html; date of access: 22 February 2022). We identified the species’ distribution across the different spatial scales using a custom script in R (see **Supplementary Data 2** for the R codes). We calculated NRI and NTI for each of the three species categories in the communities where the aliens – i) occurred alone (invasive or naturalized or introduced), and ii) cooccur with another category [invasive-naturalized – InvNat hereafter, invasive-introduced – InvInt hereafter, naturalized-introduced – NatInt hereafter]. For each community, we used two kinds of sample data – occurrence (presence only) and abundance (number of occurrences to account for the uneven number of species among the communities) of the species in the community. We also calculated the NRI and NTI anomalies [sensu (Sandel and Tsirogiannis, 2016)] across the spatial scales for the six communities. Phylogenetic anomaly is defined as the difference between the phylogenetic measures for the communities with three categories of alien species (i.e., introduced + naturalized + invasive) and that for the communities with either (single or cooccurrence) of them. In addition to analyses at the species level, we conducted all analyses as outlined above for three families (taxonomic hierarchy) which had the maximum number of alien species: Asteraceae (n=114), Fabaceae (n=90), and Poaceae (n=75) (**Figure 1**).

### 2.4 Statistical analyses

The parametric or non-parametric forms of all statistical tests were determined based on the compliance of the NRI and NTI values with the assumption of normality and homogeneity of variance. The influence of two data types (presence/abundance) on variations in phylogenetic measures of six community types (invasive, naturalized, introduced, InvNat, InvInt, NatInt) was first checked by using logistic regression models (the glm function in R).

Based on the lower AICc (the AIC score corrected for sample sizes) values of the regression models (**Supplementary Figure 1**), we selected the abundance-based phylogenetic measures for further analyses. The NRI and NTI values were compared between the community types and spatial scales by two-way ANOVA. We observed significant differences in NRI and NTI values between the community types and spatial scales (see Results); therefore, NRI and NTI values were compared between the six community types for each spatial scale separately for a better understanding. The p-values in all tests were adjusted with the Holm method. We further related patterns of phylogenetic relatedness to the bioclimatic variables to check the influence of climate on variations in NRI and NTI values. Two bioclimatic variables, namely mean annual temperature (BIO1) and annual precipitation (BIO12), at 2.5 arcminute resolution, were downloaded from the WorldClim database version 2 (Fick and Hijmans, 2017). The mean values of the bioclimatic variables were then calculated for each of the four spatial scales (7 MHTs, 16 KGCs, 34 PROs, and 47 ECOs). Logistic regression models were fitted for six community types (invasive, naturalized, introduced, InvNat, InvInt, NatInt) together and separately.

Finally, Spearman’s rank correlation (the rho coefficient, ρ) was calculated to measure the strength and direction of the relationship between the phylogenetic relatedness of the six community types at each of the four spatial scales. All statistical analyses were conducted at species and family (i.e., three selected families) levels. All statistical analyses were conducted in R; the details are provided in **Supplementary Note 2**.

## 3 Results

The 706 species (introduced = 165, naturalized = 222, invasive = 319) considered in this study belonged to the 397 genera and 94 families. The phylogenetic tree was highly resolved, with the species distributed among major angiosperm clades (**Figure 1**). Three families having the maximum number of species were Asteraceae (16.15%, n=114: introduced = 17, naturalized = 32, invasive = 65), Fabaceae (12.75%, n=90: introduced = 28, naturalized = 27, invasive = 35), and Poaceae (10.62%, n=75: introduced = 23, naturalized = 20, invasive = 32).

### 3.1 Comparison of phylogenetic measures

**Figure 3:**
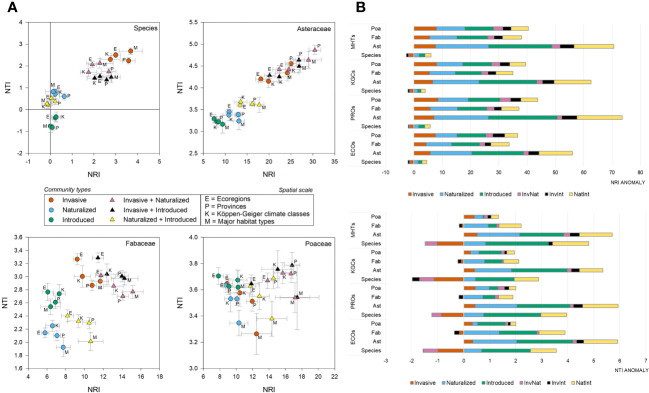
Phylogenetic position and anomalies of six community types – **(A)** The scatter plots showing the position of the six community types at four spatial scales in the two-dimensional phylogenetic spaces constructed from the net relatedness index (NRI) and nearest taxon index (NTI); **(B)** the bar plots showing the NRI and NTI anomalies for six community types at species- and family-level (Ast, Asteraceae; Fab, Fabaceae; Poa, Poaceae); at four spatial scales (ECOs, Ecoregions; KGCs, Köppen-Geiger climate classes; PROs, Provinces; MHTs, Major habitat types).

At the species level, the average NRI values [-0.14 to 3.67 with an average (± se) of 1.38 ± 0.27] were found to be higher than NTI [-0.81 to 2.68 with an average (± se) of 1.05 ± 0.21) (**Supplementary Data 3**). The phylogenetic pattern was consistent at the family level, although both the phylogenetic measures were positive and the NRI values were higher than that observed at the species level (Asteraceae: NRI=17.99 ± 1.46, NTI=3.92 ± 0.11; Fabaceae: NRI=10.01 ± 0.59, NTI=2.66 ± 0.08; Poaceae: NRI=12.40 ± 0.61, NTI=3.60 ± 0.03). In the two-dimensional phylogenetic space, the invasive, InvNat, and InvInt were placed higher than the naturalized and introduced species (**Figure 3A**). The pattern was consistent for Asteraceae and Fabaceae but not for Poaceae. At the species level, including invasive species in the community created negative phylogenetic anomalies for NRI across all spatial scales, although such a pattern was not observed at the family level (**Figure 3B**). Negative anomalies for NTI were observed at the species level and for Fabaceae at all four spatial scales (**Figure 3B**).

The two-way ANOVA revealed significant main effects of both community types and spatial scales on the NRI (community types: F_5,600_ = 84.52, p<0.001; spatial scale: F_3,600_ = 5.08, p=0.0018) and NTI (community types: F_5,600_ = 106.08, p<0.001; spatial scale: F_3,600_ = 2.85, p=0.037) values (**Table S4.1**). For the community types, the *post hoc* analysis revealed that the NRI and NTI values of the communities with the invasive species were significantly higher than that of the InvNat community (**Table 1**; **Table S4.2**). Significant differences were also observed between the NTI values of InvInt and InvNat communities as well as between Nat and NatInt communities. The phylogenetic patterns changed at the family level. The NRI and NTI values of the communities with cooccurring alien species of three different invasion statuses were generally higher than the communities where the alien species occurred alone (**Table 1**). For example, the NRI values of the InvNat and InvInt communities were significantly higher than those of the Inv communities. Among the four spatial scales, the largest NRI and NTI values were observed at PROs and ECOs, respectively (**Table 1**). A similar pattern was also observed for the three families, except for the NRI values of the Poaceae communities. Given the main effects of community types and spatial scale on phylogenetic relatedness metrics, we further separately compared the NRI and NTI values between the six community types for each spatial scale (**Table S5.1**).

**Table 1:** Estimates (average ± standard error) of the two phylogenetic measures [net relatedness index (NRI) and nearest taxon index (NTI)] for six communities, at species- and family levels, and at four spatial scales.

	Community types	N	NRI ± se	NTI ± se	Spatial scales	N	NRI ± se	NTI ± se
Species	Inv	104	3.196^a^ ± 0.16	2.398^b^ ± 0.08	ECOs	282	1.286^b^ ± 0.09	1.180^b^ ± 0.08
	Nat	104	0.363 ± 0.10	0.734* ± 0.09	PROs	204	1.635^a^ ± 0.14	0.920^a^ ± 0.09
	Int	104	0.179 ± 0.08	-0.535 ± 0.09	KGCs	96	1.158^b^ ± 0.16	1.009 ± 0.12
	InvNat	104	2.167^b^ ± 0.16	1.910^a^ ± 0.09	MHTs	42	1.456 ± 0.28	1.109 ± 0.20
	InvInt	104	2.371 ± 0.13	1.461^b^ ± 0.08				
	NatInt	104	0.074 ± 0.08	0.413^#^ ± 0.07				
Asteraceae	Inv	103	21.095^a^ ± 0.84	4.319^a ±^0 0.06	ECOs	250	15.989^a^ ± 0.55	3.926^a^ ± 0.04
	Nat	94	11.800* ± 0.44	3.400* ± 0.04	PROs	201	20.165^b^ ± 0.69	4.063^b^ ± 0.06
	Int	83	8.087^#^ ± 0.29	3.247^#^ ± 0.05	KGCs	91	16.962^a^ ± 1.02	3.883^a^ ± 0.07
	InvNat	103	25.808^b^ ± 0.98	4.582^b^ ± 0.06	MHTs	42	20.059^b^ ± 1.36	3.916 ± 0.12
	InvInt	103	23.146 ± 0.83	4.411 ± 0.05				
	NatInt	98	14.704^#^ ± 0.48	3.630^#^ ± 0.04				
Fabaceae	Inv	104	9.985^a^ ± 0.29	3.073* ± 0.06	ECOs	248	9.063^a^ ± 0.27	2.851^a^ ± 0.04
	Nat	94	6.517 ± 0.26	2.129 ± 0.04	PROs	201	10.604^b^ ± 0.31	2.597^b^ ± 0.04
	Int	79	6.625 ± 0.33	2.680^#^ ± 0.06	KGCs	92	9.861 ± 0.47	2.705 ± 0.06
	InvNat	104	12.947^b^ ± 0.39	2.872^a^ ± 0.05	MHTs	42	10.970^b^ ± 0.66	2.525^b^ ± 0.08
	InvInt	104	12.599^b^ ± 0.42	3.136^b^ ± 0.05				
	NatInt	98	9.346 ± 0.44	2.324^#^ ± 0.05				
Poaceae	Inv	100	10.437^b^* ± 0.45	3.563 ± 0.05	ECOs	258	10.634^a^ ± 0.30	3.653^a^ ± 0.03
	Nat	101	9.382 ± 0.26	3.564 ± 0.03	PROs	203	13.212^b^ ± 0.35	3.645^a^ ± 0.03
	Int	87	8.880 ± 0.40	3.663 ± 0.04	KGCs	93	12.318 ^b^ ± 0.58	3.635^a^ ± 0.04
	InvNat	103	15.281* ± 0.44	3.687 ± 0.04	MHTs	42	13.666 ^b^ ± 0.84	3.448^b^ ± 0.06
	InvInt	103	14.365^a^ ± 0.65	3.702 ± 0.05				
	NatInt	102	13.018^#^ ± 0.45	3.619 ± 0.04				

Community types – Inv, Invasive; Nat, Naturalized; Int, Introduced; InvNat, Invasive + Naturalized; InvInt, Invasive + Introduced; NatInt, Naturalized + Introduced; Different letters and symbols indicate significant differences at p<0.05 level.

**Figure 4:**
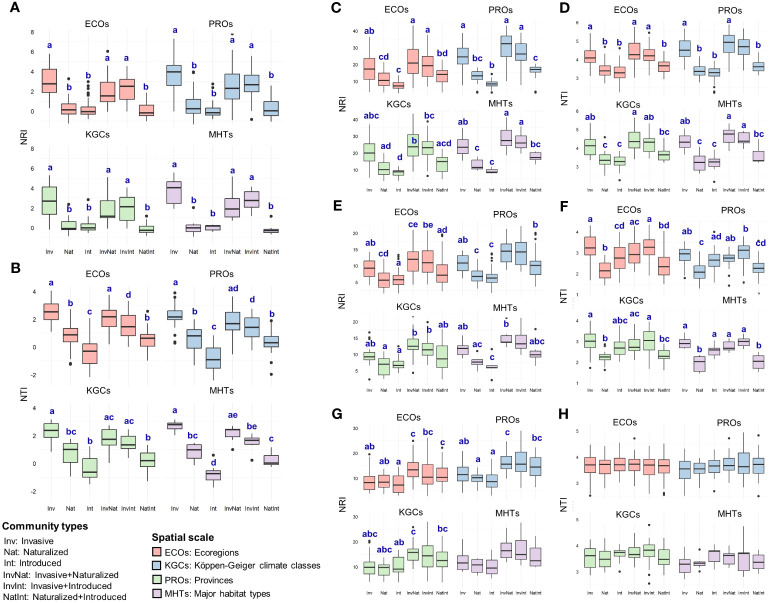
Comparative assessment of the net relatedness index (NRI) and nearest taxon index (NTI) between six community types at – **(A, B)** species level and **(C-H)** family level [**(C, D)** Asteraceae; **(E, F)**: Fabaceae; **(G, H)** Poaceae], at four spatial scales (ECOs, Ecoregions; PROs, Provinces; KGCs, Köppen-Geiger climate classes; MHTs, Major habitat types) separately. Different letters on the box plots indicate significant differences (at p<0.05 level) between the community types. The box plots without letters on top indicate no significant differences between the community types. The statistical estimates (F-values, t-statistics, degrees of freedom, and actual p-values) are given in **Supplementary Data 5**.

Across the spatial scales, NRI values of the communities with the invasive species (alone or in combination) were significantly higher than that of the naturalized and introduced alien species (**Figure 4A**, **Table S5.2**). No significant differences were observed between the NRI values of the naturalized and introduced species (alone or in combination). Similar to the pattern observed for NRI, the NTI values of the communities with the invasive species (alone or in combination) were significantly higher than that of the naturalized and introduced alien species (**Figure 4B**, **Table S5.2**). In addition, naturalized species showed significantly higher NTI values than that of the introduced species (occurring alone) across three of the four spatial scales (ECOs, PROs, and MHTs) (**Figure 4B**, **Table S5.2**). The phylogenetic pattern was different at the family level than at the species level and varied between families (**Figure 4C-H**, **Table S5.2**). For Asteraceae (**Figure 4C, D**) and Fabaceae (**Figure 4E, F**), the phylogenetic measures of the communities having the invasive species (either occurring alone or in combination) were higher than that of the communities having the naturalized and introduced species across all spatial scales. However, the pattern of significant differences varied from the pattern observed at the family level and between the two families (**Table S5.2**). For the family Poaceae, very few significant differences were observed between the NRI values of the six community types (**Figure 4G**), and no significant differences in the NTI values of the six communities were observed across the spatial scales (**Figure 4H**).

### 3.2 Influence of bioclimatic variables on phylogenetic measures

**Figure 5:**
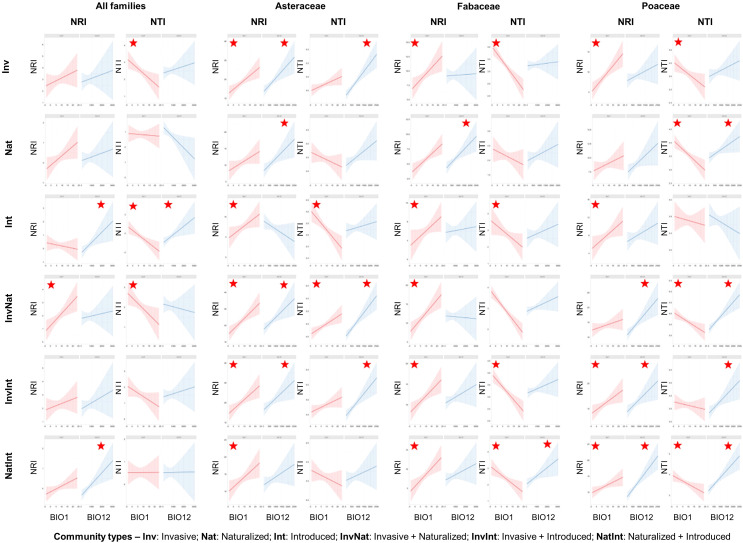
Scatterplots of regression analysis outputs showing the influence of two bioclimatic variables (annual mean temperature: BIO1 and annual precipitation: BIO12) on the two phylogenetic measures: net relatedness index (NRI) and nearest taxon index (NTI). The regression analyses were conducted for six community types, at species- and family levels, and at four spatial scales (the outputs of the finest spatial scale, i.e., the Ecoregion scale, are shown here). The red stars indicate significant relationships at p<0.05 level. The statistical estimates of all regression models (t statistics, degrees of freedom, and actual p-values) are provided in **Supplementary Data 6**.

The regression models revealed that the NRI values were generally positively related to the bioclimatic variables, while more negative relationships were observed between the NTI values and the bioclimatic variables (**Supplementary Data 6**). The pattern was more evident at the finest spatial scale (ECOs), where the NRI values were mostly positively related to BIO1 (5 out of 6 communities, with the InvNat community showing significance at p=0.05 level at the ecoregion scale) and BIO12 (all six community types, with the Int and NatInt communities showing significance) (**Figure 5**). On the other hand, the NTI values were mostly negatively related to BIO1 (all six communities, with the Inv, Int, and InvNat communities showing significance). Compared to the species level, more significant positive relationships between NRI and the two bioclimatic variables were observed at the family level at this scale (ECOs). Among the three families, the maximum number of significant negative relationships between NTI and BIO1 were observed in Fabaceae (Inv, Int, InvInt, and NatInt) and Poaceae ((Inv, Nat, InvNat, and NatInt) communities (**Figure 5**).

### 3.3 Correlation of phylogenetic measures between community types

**Figure 6:**
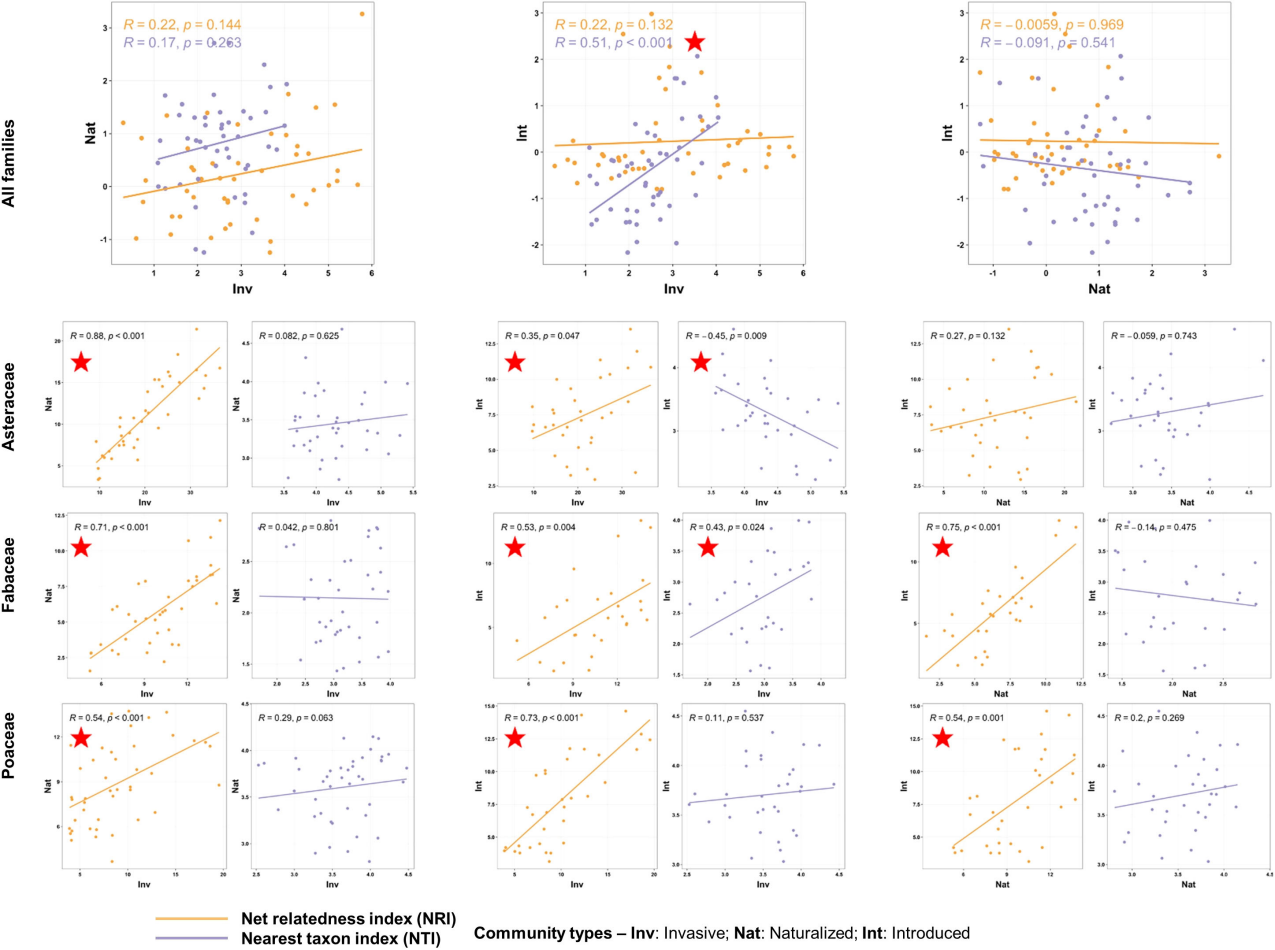
Scatterplots showing correlations of the phylogenetic measures [net relatedness index (NRI) and nearest taxon index (NTI)] between the communities with alien species of three invasion categories occurring alone at the finest spatial scale, i.e., the Ecoregion scale. The correlation analyses were conducted at the species level (top row) and family level (bottom rows). At the family level, correlations of the NRI and NTI values between the communities are presented in separate plots. The rho and p-values are mentioned, and the red stars indicate significant correlations at p<0.05. The statistical estimates (rho values, actual p-values, and sample sizes) of the correlations of the phylogenetic measures between all six community types at four spatial scales are provided in **Supplementary Data 7**.

The correlations of the phylogenetic measures between the three species categories (invasive, naturalized, and introduced) were mostly non-significant across the spatial scale (**Supplementary Data 7**). We only found a positive correlation between the NTI values of the invasive and introduced species communities at the ecoregion scale (ρ = -0.508, p = 0.0003; **Figure 6**) and between both phylogenetic measures of the invasive and naturalized species at the provincial scale (PROs, NRI: ρ = 0.588, p = 0.0003; NTI: ρ = 0.378, p = 0.028). The phylogenetic measures of the naturalized and introduced species were mostly negatively correlated across the spatial scales. However, at the family level and the finest spatial scale (ECOs), the NRI values of the three communities were positively correlated (except for the NRI values of the Poaceae naturalized and introduced communities, **Figure 6**). Significant correlations were also observed between the NTI values of the invasive and introduced communities of the family Asteraceae (ρ = -0.45, p = 0.009) and Fabaceae (ρ = 0.428, p = 0.024) (**Figure 6**).

## 4 Discussion

In this study, we tested three hypotheses related to the variation in phylogenetic relatedness between alien species of three invasion categories at four spatial and two taxonomic scales. The findings of our study supported these hypotheses. We observed more clustered assemblages at finer spatial scales for all three alien species categories at species and family levels. Significantly higher NRI and NTI were observed at provincial (PROs) and ecoregion (ECOs) scales, respectively. These findings supported our first hypothesis, based on the concept that closely related species can respond more similarly to the environmental variation that is more noticeable at a finer spatial scale (Procheş et al., 2008).

The second hypothesis, i.e., the phylogenetic measures will vary between the alien species of three categories, with the invasive aliens showing the most clustered assemblage, was supported by multiple findings of this study. Firstly, the presence of introduced alien species in the community caused a clustered assemblage when the NRI values were considered (positive NRI) and an overdispersed assemblage when considering the NTI values (negative NTI). In contrast, positive NTI values were observed for the invasive and naturalized alien species. These findings indicate that although the existing lineages of the introduced species may lead to small mean pairwise distances, they do not always have a close relative present in the community. The positive NTI values for the invasive and naturalized species, on the other hand, resonates with the pre-adaptation hypothesis, which posits that a close relative in the community may help the successful naturalization and invasion of the introduced alien species (Ricciardi and Mottiar, 2006). Secondly, the communities with invasive species had significantly higher NRI and NTI values across the spatial scales, thus being concordant with the previous studies (Zhang et al., 2021; Qian et al., 2022b). Including invasive species created a more clustered community, as evident from the negative NRI and NTI anomalies. Besides, the NRI values were not significantly different between the naturalized and introduced species. This phylogenetic pattern is indicative that invasive species occupy a different phylogenetic niche than the naturalized and introduced species, as also evident from the two-dimensional phylogenetic space. This finding is consistent with a previous observation that established alien species need to be phylogenetically different to become invasive (Divisek et al., 2018). The communities with naturalized and introduced species were found to have high NTI and negative NTI values, respectively, across all spatial scales. Therefore, for successful establishment and naturalization, the introduced species should have an immediate relative present in the community; however, the presence of invasive aliens could facilitate the establishment of other invasive species due to their similar responses to the environment. This finding provides the phylogenetic basis of the invasional meltdown hypothesis (IMH), which posits that positive interactions among invaders may promote secondary invasions (Green et al., 2011).

We found few significant correlations between the phylogenetic relatedness metrics of the three species categories. The regression analysis also revealed the influence of community types and bioclimatic variables on the NRI and NTI values, more prominently at the ecoregion (ECOs) scale. Given that environmental filtering often drives phylogenetic clustering (Webb et al., 2002), these findings indicate that different environmental factors may influence the alien species at different stages of the invasion continuum. In general, the phylogenetic relatedness of the alien species in China was more influenced by annual mean temperature (BIO1) than the annual precipitation (BIO12). With the decrease in temperature, the NTI values increased, as evident from the negative relationship between NTI and BIO1. This finding implies that with increased environmental stress (e.g., low temperature), phylogenetic clustering increased between species sharing similar environmental space (high NTI). A similar influence of cold stress on phylogenetic clustering has been found in other studies on plants (Li et al., 2014), animals (Pellissier et al., 2013), and microbes (Wang et al., 2012). When we analyzed the influence of the environment on the phylogenetic measures for three alien species categories separately, the NRI and NTI values of the communities with the invasive aliens were found to be more influenced by temperature (BIO1). At the other end of the continuum, precipitation (BIO12) influenced the introduced aliens’ phylogenetic measures more. Moreover, compared to the introduced species, more clustered NTIs were observed for the invasive species in response to decreasing temperature when they occurred alone and combined with the naturalized species. This phylogenetic pattern suggests a more specialized response of the invasive aliens to the environmental filters than the naturalized and introduced species, possibly due to a higher level of trait plasticity, which provides the invasives a greater tolerance to different environmental conditions (Gallagher et al., 2015). It is also possible that the closely related invasive species can retain their niche-related traits through evolutionary history and, therefore, can respond similarly in the environmental space. This finding is thus consistent with the phylogenetic niche conservatism hypothesis (Crisp and Cook, 2012), which has been reported previously for both plant (Liu et al., 2015) and animal species (Cooper et al., 2011).

The phylogenetic pattern varied at the family level from that observed at the species level, thereby supporting our third hypothesis. Both the phylogenetic measures were positive for three alien species categories. With the decrease in the phylogenetic resolution, the species of the same family respond to the environmental gradients more similarly, which can explain the clustered assemblage within families. Indeed, compared to the species level, we observed more significant correlations between the NRI values of the three alien species categories at the family level. In each of the three families, the correlation analysis revealed that the NRI values of the three species categories were positively correlated mainly at finer environmental scales (ECOs). This pattern can be explained based on the similar response of all possible species pairs of the same family to the environmental filtering process (Webb et al., 2002). The shared response to environmental filters within families is also supported by the observation that the bioclimatic variables more influenced NRI values; the pattern in contrast to that observed at the species level where the environmental variables more influenced variation in NTI values. These findings provide evidence of the varied environmental influence at different levels of the phylogenetic tree (Qian et al., 2022a). Besides, the NRI values were higher than the NTIs for all three families, creating a more clustered NRI and less clustered NTI. These findings support the observation that NRI tends to be driven by structure deep in the tree, while patterns near the tip more strongly influence NTI (Mazel et al., 2016). Overall, the phylogenetic patterns observed at species and family levels in this study are consistent with a previous meta-analysis of phylogenetic relatedness patterns (Vamosi et al., 2009).

However, the phylogenetic pattern was not always consistent across families. The invasive species of Asteraceae, either occurring alone or in combination with the other two alien species categories, showed a significantly stronger clustered community assemblage. This pattern became weaker and dependent on the spatial scale in Fabaceae, where significantly higher NRI and NTI values were only observed when the invasives cooccurred with the naturalized and introduced species. We observed no significant differences in the phylogenetic measures in Poaceae between the three species categories. The variations in phylogenetic patterns between families can be explained based on the families’ different evolutionary histories (Harris and Davies, 2016) and the species’ different life history traits, which are correlated with the rate of molecular evolution (Smith and Donoghue, 2008). For example, previous studies have found that plant growth form has an inherent phylogenetic signal (Kerkhoff et al., 2006). On the one hand, the majority of the Asteraceae species included in this study were herbs (113 out of 114 species; see **Supplementary Data 1**), which might have undergone a higher rate of molecular evolution than shrubs and trees (Smith and Donoghue, 2008), thereby creating the observed phylogenetic signal. On the other hand, the three alien species groups of the Poaceae family might have evolved enough times independently to obscure any clear signal of phylogenetic clustering. Besides, the phylogenetic structure is heavily influenced by polyploidy (Rothfels, 2021), which occurs more often in Poaceae than in any other angiosperm family. Nearly 80% of grass species are supposed to have undergone a ploidy change at some point in their evolution (Huang et al., 2022), thereby obscuring the phylogenetic signal. The variations in phylogenetic patterns between Asteraceae and Poaceae can also be generated due to the uneven number of alien species in three invasion categories. Compared to the higher number of invasive aliens in Asteraceae (65 out of 114 amounting to 57.02%), the relatively balanced proportions of alien species of three invasion categories in Poaceae (32 invasive aliens, 20 naturalized aliens, and 23 introduced aliens) may also cause the absence of clear phylogenetic signal.

Similarly, the influence of the environmental filtering process on phylogenetic relatedness was not consistent across the three families considered in this study. The effect of cold stress leading to increased phylogenetic clustering (higher NTI values with a decrease in temperature), as observed at the species level and for Fabaceae and Poaceae families, was not observed for Asteraceae invasive and naturalized species. In addition, the correlation analysis revealed a significant positive correlation of both phylogenetic measures between invasive and naturalized aliens of Asteraceae across all spatial scales. These findings indicate that the invasives and naturalized species of the family Asteraceae might have been subjected to the same environmental filtering pressure. This phylogeographic pattern was not observed in Fabaceae, where, for example, the NRI and NTI values of the invasive and naturalized species showed positive and negative relationships, respectively, with annual precipitation. The different growth forms of the Fabaceae aliens (47 herbs, 29 shrubs, and 14 trees; see **Supplementary Data 1**), compared to the herbaceous growth form of the majority of the Asteraceae species, could explain the varied response of different categories of alien species to the environmental filtering process. Such details of the phylogenetic relationship are obscured with increased phylogenetic extent (e.g., at the tip of the tree) and spatial scale of the investigation by combining species with different response abilities (e.g., Asteraceae and Fabaceae) to environmental filtering processes (Weigelt et al., 2015).

## 5 Conclusions and future directions

In conclusion, our study showed that the phylogenetic relatedness of the alien species varies across the invasion continuum. The pattern depends greatly on the environment and spatial scale of investigation. The invasive species community showed the most clustered assemblage, irrespective of the spatial scales. The influence of the environmental filtering process also varied between families, more so at finer spatial scales, although there was a consistent tendency for more clustered NRI for all three species groups. Overall, our study highlights the importance of assessing hierarchical phylogenetic patterns of alien plant species of different invasion stages across spatial and taxonomic scales. We have also identified specific scopes of improvements that can further advance our understanding of the phylogenetic changes across the invasion process. Firstly, the uneven number of species across different communities and taxonomic scales might influence the comparative assessment of the phylogenetic measures. However, using abundance-weighted phylogenetic measures addressed this problem to some extent. Secondly, the occurrence data used in this study to estimate the phylogenetic metrics were collected from GBIF and NSII, which often cannot adequately represent the actual distribution of the species in China (Qian et al., 2018). In addition, the availability of new information on the alien flora of China [e.g., (Lin et al., 2022)] can change the current findings. Thirdly, we selected the families with the maximum number of alien species. The phylogenetic pattern at the taxonomic scale may also vary depending on the choice of families due to their different evolutionary histories. Future studies can gain novel insights into the phylogenetic pattern by including families having the maximum number of invasive and/or naturalized and/or introduced species (e.g., families of genera, like *Amaranthus*, *Ipomoea*, *and Solanum*, with many invasive species from this study). Finally, our study also indicated that plant functional traits could influence phylogenetic patterns in response to the environmental filtering process; therefore, relating phylogenetic patterns with functional traits of alien plant species in future studies can provide novel insights into the invasion process. As the phylogenetic structure is often determined by interacting species present in each community, we suggest future phylogenetic analysis should consider the combination of native and alien species at multiple spatial and taxonomic scales to understand better the dynamics of recipient community changes across the invasion continuum.

## Data availability statement

The original contributions presented in the study are included in the article/**Supplementary Material**. Further inquiries can be directed to the corresponding author.

## Author contributions

AB and YH conceived and planned the project. HF, XL, YL, JW, MY, and HP collected the data. AB, FT, HF, and NZ analyzed the data. AB, FT, NZ, and YH contributed to the interpretation of the results. AB wrote the first draft of the manuscript. All authors contributed to the article and approved the submitted version.

## Supplementary material

Supplementary Data Sheet 1 Species details and occurrence data.

Supplementary Data Sheet 2 R code and associated data.

Supplementary Data Sheet 3 Descriptive estimates of phylogenetic measures.

Supplementary Data Sheet 4 Statistical estimates of comparative analysis across spatial scales.

Supplementary Data Sheet 5 Statistical estimates of comparative analysis at individual spatial scale.

Supplementary Data Sheet 7 Statistical estimates of correlation analysis.

## References

[B1] Axmacher J. C. Sang W. (2013). Plant invasions in China – challenges and chances. PloS One 8, e64173. doi: 10.1371/journal.pone.0064173 23691164 PMC3653845

[B2] Brown J. L. (2014). SDMtoolbox: a python-based GIS toolkit for landscape genetic, biogeographic and species distribution model analyses. Methods Ecol. Evol. 5, 694–700. doi: 10.1111/2041-210X.12200 PMC5721907 29230356

[B3] Carvallo G. O. Teillier S. Castro S. A. Figueroa J. A. (2014). The phylogenetic properties of native- and exotic-dominated plant communities. Austral Ecol. 39, 304–312. doi: 10.1111/aec.12079

[B4] Chamberlain S. Szöcs E. (2013). taxize: taxonomic search and retrieval in R [version 2; peer review: 3 approved]. F1000Research 2, 191. doi: 10.12688/f1000research.2-191.v1 24555091 PMC3901538

[B5] Cooper N. Freckleton R. P. Jetz W. (2011). Phylogenetic conservatism of environmental niches in mammals. Proc. R. Soc. B.: Biol. Sci. 278, 2384–2391. doi: 10.1098/rspb.2010.2207 PMC3119006 21208967

[B6] Crisp M. D. Cook L. G. (2012). Phylogenetic niche conservatism: what are the underlying evolutionary and ecological causes? New Phytol. 196, 681–694. doi: 10.1111/j.1469-8137.2012.04298.x 22943495

[B7] Daehler C. C. (2001). Darwin’s naturalization hypothesis revisited. Am. Nat. 158, 324–330. doi: 10.1086/321316 18707328

[B8] Divisek J. Chytrý M. Beckage B. Gotelli N. J. Lososová Z. Pyšek P.. (2018). Similarity of introduced plant species to native ones facilitates naturalization, but differences enhance invasion success. Nat. Commun. 9, 4631. doi: 10.1038/s41467-018-06995-4 30401825 PMC6219509

[B9] Donoghue M. J. (2008). A phylogenetic perspective on the distribution of plant diversity. Proc. Natl. Acad. Sci. 105, 11549–11555. doi: 10.1073/pnas.0801962105 18695216 PMC2556411

[B10] ESRI (2011). ArcGIS Desktop: Release 10 (Redlands, CA: Environmental Systems Research Institute).

[B11] Fick S. E. Hijmans R. J. (2017). WorldClim 2: new 1-km spatial resolution climate surfaces for global land areas. Int. J. Climatol. 37, 4302–4315. doi: 10.1002/joc.5086

[B12] Gallagher R. V. Randall R. P. Leishman M. R. (2015). Trait differences between naturalized and invasive plant species independent of residence time and phylogeny. Conserv. Biol. 29, 360–369. doi: 10.1111/cobi.12399 25369762 PMC4405095

[B13] GBIF.Org (2022). GBIF Occurrence Download https://doi.org/10.15468/dl.9qfsgk.

[B14] Green P. T. O’dowd D. J. Abbott K. L. Jeffery M. Retallick K. Mac Nally R. (2011). Invasional meltdown: invader-invader mutualism facilitates a secondary invasion. Ecology 92, 1758–1768. doi: 10.1890/11-0050.1 21939072

[B15] Harris L. W. Davies T. J. (2016). A complete fossil-calibrated phylogeny of seed plant families as a tool for comparative analyses: Testing the ‘Time for Speciation’ hypothesis. PloS One 11, e0162907. doi: 10.1371/journal.pone.0162907 27706173 PMC5051821

[B16] Hodgins K. A. Bock D. G. Rieseberg L. H. (2018). Trait evolution in invasive species. Annu. Plant Rev. Online 1, 459–496. doi: 10.1002/9781119312994.apr0643

[B17] Huang W. Zhang L. Columbus J. T. Hu Y. Zhao Y. Tang L.. (2022). A well-supported nuclear phylogeny of Poaceae and implications for the evolution of C_4_ photosynthesis. Mol. Plant 15, 755–777. doi: 10.1016/j.molp.2022.01.015 35093593

[B18] Jiang H. Fan Q. Li J.-T. Shi S. Li S.-P. Liao W.-B.. (2011). Naturalization of alien plants in China. Biodiversity Conserv. 20, 1545–1556. doi: 10.1007/s10531-011-0044-x

[B19] Jin Y. Qian H. (2019). V.PhyloMaker: an R package that can generate very large phylogenies for vascular plants. Ecography 42, 1353–1359. doi: 10.1111/ecog.04434 PMC9363651 35967255

[B20] Kerkhoff A. J. Fagan W. F. Elser J. J. Enquist B. J. (2006). Phylogenetic and growth form variation in the scaling of nitrogen and phosphorus in the seed plants. Am. Nat. 168, E103–E122. doi: 10.1086/507879 17004214

[B21] Kindt R. (2020). WorldFlora: An R package for exact and fuzzy matching of plant names against the World Flora Online taxonomic backbone data. Appl. Plant Sci. 8, e11388. doi: 10.1002/aps3.11388 33014632 PMC7526431

[B22] Letcher S. G. (2010). Phylogenetic structure of angiosperm communities during tropical forest succession. Proc. R. Soc. B.: Biol. Sci. 277, 97–104. doi: 10.1098/rspb.2009.0865 PMC2842617 19801375

[B23] Letunic I. Bork P. (2016). Interactive tree of life (iTOL) v3: an online tool for the display and annotation of phylogenetic and other trees. Nucleic Acids Res. 44, W242–W245. doi: 10.1093/nar/gkw290 27095192 PMC4987883

[B24] Li X.-H. Zhu X.-X. Niu Y. Sun H. (2014). Phylogenetic clustering and overdispersion for alpine plants along elevational gradient in the Hengduan Mountains Region, southwest China. J. Systematics Evol. 52, 280–288. doi: 10.1111/jse.12027

[B25] Lin Q. Xiao C. Ma J. (2022). A dataset on catalogue of alien plants in China. Biodiversity Sci. 30, 22127. doi: 10.17520/biods.2022127

[B26] Liu C. Wolter C. Xian W. Jeschke J. M. (2020). Most invasive species largely conserve their climatic niche. Proc. Natl. Acad. Sci. United States America 117, 23643–23651. doi: 10.1073/pnas.2004289117 PMC7519298 32883880

[B27] Liu H. Xu Q. He P. Santiago L. S. Yang K. Ye Q. (2015). Strong phylogenetic signals and phylogenetic niche conservatism in ecophysiological traits across divergent lineages of Magnoliaceae. Sci. Rep. 5, 12246. doi: 10.1038/srep12246 26179320 PMC4503962

[B28] Ma J. Li H. (2018). The Checklist of the Alien Invasive Plants in China (Beijing: Higher Education Press).

[B29] Maurel N. Hanspach J. Kühn I. Pyšek P. Van Kleunen M. (2016). Introduction bias affects relationships between the characteristics of ornamental alien plants and their naturalization success. Global Ecol. Biogeography 25, 1500–1509. doi: 10.1111/geb.12520

[B30] Mazel F. Davies T. J. Gallien L. Renaud J. Groussin M. Münkemüller T.. (2016). Influence of tree shape and evolutionary time-scale on phylogenetic diversity metrics. Ecography 39, 913–920. doi: 10.1111/ecog.01694 27713599 PMC5049687

[B31] Moodley D. Geerts S. Richardson D. M. Wilson J. R. U. (2013). Different traits determine introduction, naturalization and invasion success in woody plants: Proteaceae as a test case. PloS One 8, e75078. doi: 10.1371/journal.pone.0075078 24086442 PMC3782508

[B32] NSII (2021). http://www.nsii.org.cn/2017/home-en.php.

[B33] Omer A. Fristoe T. Yang Q. Razanajatovo M. Weigelt P. Kreft H.. (2022). The role of phylogenetic relatedness on alien plant success depends on the stage of invasion. Nat. Plants 8, 906–914. doi: 10.1038/s41477-022-01216-9 35953709

[B34] Pellissier L. Alvarez N. Espíndola A. Pottier J. Dubuis A. Pradervand J.-N.. (2013). Phylogenetic alpha and beta diversities of butterfly communities correlate with climate in the western Swiss Alps. Ecography 36, 541–550. doi: 10.1111/j.1600-0587.2012.07716.x

[B35] Prentis P. J. Wilson J. R. Dormontt E. E. Richardson D. M. Lowe A. J. (2008). Adaptive evolution in invasive species. Trends Plant Sci. 13, 288–294. doi: 10.1016/j.tplants.2008.03.004 18467157

[B36] Procheş Ş. Forest F. Jose S. De Dominicis M. Ramdhani S. Wiggill T. (2015). How do alien plants fit in the space-phylogeny matrix? PloS One 10, e0123238. doi: 10.1371/journal.pone.0123238 25893962 PMC4403803

[B37] Procheş Ş. Wilson J. R. U. Richardson D. M. Rejmánek M. (2008). Searching for phylogenetic pattern in biological invasions. Global Ecol. Biogeography 17, 5–10. doi: 10.1111/j.1466-8238.2007.00333.x

[B38] Pyron R. A. Costa G. C. Patten M. A. Burbrink F. T. (2015). Phylogenetic niche conservatism and the evolutionary basis of ecological speciation. Biol. Rev. Cambridge Philos. Soc. 90, 1248–1262. doi: 10.1111/brv.12154 25428167

[B39] Qian H. Deng T. Beck J. Sun H. Xiao C. Jin Y.. (2018). Incomplete species lists derived from global and regional specimen-record databases affect macroecological analyses: A case study on the vascular plants of China. J. Biogeography 45, 2718–2729. doi: 10.1111/jbi.13462

[B40] Qian H. Jin Y. (2015). An updated megaphylogeny of plants, a tool for generating plant phylogenies and an analysis of phylogenetic community structure. J. Plant Ecol. 9, 233–239. doi: 10.1093/jpe/rtv047

[B41] Qian H. Leprieur F. Jin Y. Wang X. Deng T. (2022a). Influence of phylogenetic scale on the relationships of taxonomic and phylogenetic turnovers with environment for angiosperms in China. Ecol. Evol. 12, e8544–e8544. doi: 10.1002/ece3.8544 35154648 PMC8821769

[B42] Qian H. Qian S. Sandel B. (2022b). Phylogenetic structure of alien and native species in regional plant assemblages across China: Testing niche conservatism hypothesis versus niche convergence hypothesis. Global Ecol. Biogeography 31, 1864–1876. doi: 10.1111/geb.13566

[B43] Qian H. Rejmánek M. Qian S. (2022c). Are invasive species a phylogenetically clustered subset of naturalized species in regional floras? A case study for flowering plants in China. Diversity Distributions 28, 2084–2093. doi: 10.1111/ddi.13608

[B44] Qian H. Sandel B. (2017). Phylogenetic relatedness of native and exotic plants along climate gradients in California, USA. Diversity Distributions 23, 1323–1333. doi: 10.1111/ddi.12620

[B45] R Core Team (2020). R: A Language and Environment for Statistical Computing (Vienna, Austria: R Foundation for Statistical Computing).

[B46] Ricciardi A. Mottiar M. (2006). Does Darwin’s naturalization hypothesis explain fish invasions? Biol. Invasions 8, 1403–1407. doi: 10.1007/s10530-006-0005-6

[B47] Rothfels C. J. (2021). Polyploid phylogenetics. New Phytol. 230, 66–72. doi: 10.1111/nph.17105 33491778

[B48] Sandel B. Tsirogiannis C. (2016). Species introductions and the phylogenetic and functional structure of California’s grasses. Ecology 97, 472–483. doi: 10.1890/15-0220.1 27145621

[B49] Simberloff D. Martin J. L. Genovesi P. Maris V. Wardle D. A. Aronson J.. (2013). Impacts of biological invasions: what’s what and the way forward. Trends Ecol. Evol. 28, 58–66. doi: 10.1016/j.tree.2012.07.013 22889499

[B50] Simberloff D. Von Holle B. (1999). Positive interactions of nonindigenous species: invasional meltdown? Biol. Invasions 1, 21–32. doi: 10.1023/A:1010086329619

[B51] Smith S. A. Brown J. W. (2018). Constructing a broadly inclusive seed plant phylogeny. Am. J. Bot. 105, 302–314. doi: 10.1002/ajb2.1019 29746720

[B52] Smith S. A. Donoghue M. J. (2008). Rates of molecular evolution are linked to life history in flowering plants. Science 322, 86–89. doi: 10.1126/science.1163197 18832643

[B53] Thuiller W. Gallien L. Boulangeat I. De Bello F. Münkemüller T. Roquet C.. (2010). Resolving Darwin’s naturalization conundrum: a quest for evidence. Diversity Distributions 16, 461–475. doi: 10.1111/j.1472-4642.2010.00645.x

[B54] Vamosi S. M. Heard S. B. Vamosi J. C. Webb C. O. (2009). Emerging patterns in the comparative analysis of phylogenetic community structure. Mol. Ecol. 18, 572–592. doi: 10.1111/j.1365-294X.2008.04001.x 19037898

[B55] Wang J. Soininen J. He J. Shen J. (2012). Phylogenetic clustering increases with elevation for microbes. Environ. Microbiol. Rep. 4, 217–226. doi: 10.1111/j.1758-2229.2011.00324.x 23757276

[B56] Webb C. O. (2000). Exploring the phylogenetic structure of ecological communities: An example for rain forest trees. Am. Nat. 156, 145–155. doi: 10.1086/303378 10856198

[B57] Webb C. O. Ackerly D. D. Kembel S. W. (2008). Phylocom: software for the analysis of phylogenetic community structure and trait evolution. Bioinformatics 24, 2098–2100. doi: 10.1093/bioinformatics/btn358 18678590

[B58] Webb C. O. Ackerly D. D. Mcpeek M. A. Donoghue M. J. (2002). Phylogenies and community ecology. Annu. Rev. Ecol. Evol. Systematics 33, 475–505. doi: 10.1146/annurev.ecolsys.33.010802.150448

[B59] Weigelt P. Daniel Kissling W. Kisel Y. Fritz S. A. Karger D. N. Kessler M.. (2015). Global patterns and drivers of phylogenetic structure in island floras. Sci. Rep. 5, 12213. doi: 10.1038/srep12213 26198002 PMC4510489

[B60] Wiens J. J. Donoghue M. J. (2004). Historical biogeography, ecology and species richness. Trends Ecol. Evol. 19, 639–644. doi: 10.1016/j.tree.2004.09.011 16701326

[B61] Zhang A. Hu X. Yao S. Yu M. Ying Z. (2021). Alien, naturalized and invasive plants in China. Plants 10, 2241. doi: 10.3390/plants10112241 34834604 PMC8620670

[B62] Zhao C. Liu Q. Li F. Wong L. J. Pagad S. (2020) Global Register of Introduced and Invasive Species - China. Version 1.3. Invasive Species Specialist Group ISSG. Checklist dataset (Accessed 2022-02-20).

