# Supplementary material for: Invasive alien plants are phylogenetically distinct from other alien species across spatial and taxonomic scales in China
Source: Front Plant Sci. 2023 Sep 7;14:1075344. doi: 10.3389/fpls.2023.1075344 (PMC10513447; doi:10.3389/fpls.2023.1075344)
Supplement: Supplementary file 2 [file Table_1.docx]

Supplementary Material

# Supplementary Note 1: Categorization of alien flora in China

We developed a multistep pipeline for categorization of alien plant species of China. In the first step, three nationwide checklists, viz. naturalized alien plants of China [n = 861; ([Jiang et al., 2011](#_ENREF_6))], The checklist of the Alien Invasive Plants in China [n = 464; ([Ma and Li, 2018](#_ENREF_8))], and the Global Register of Introduced and Invasive Species (GRIIS) - China Version 1.3 [n = 459; ([Zhao et al., 2020](#_ENREF_19))], were consulted to create a comprehensive list of alien plant species of China. To remove ambiguity and orthographical errors in the plant nomenclature, automated standardization of taxonomic names was conducted using the *WorldFlora* package ([Kindt, 2020](#_ENREF_7)) in R version 4.0.2 ([R Core Team, 2020](#_ENREF_14)) (hereafter, R). This package validates the species names against a static version of the World Flora Online (WFO) taxonomic backbone data, which is actively curated by global experts based on The PlantList backbone (<http://www.theplantlist.org/>), and therefore provides the most updated and comprehensive taxonomic reference of vascular plants ([Borsch et al., 2020](#_ENREF_1)). The list was further cleaned by removing duplicates and synonyms, and the infraspecific taxa and artificially hybridized species were not considered further.

The selected plants (n=912) were subjected to a two-step verification process involving the three national-level checklists mentioned above and two global databases to determine their – i) origin status (i.e., is the species native or alien to China) and ii) invasion status (i.e., what is the degree of naturalization of the species) ([Pyšek et al., 2004](#_ENREF_12)) in China. We chose two global databases, namely the Plants of the World Online (POWO; <https://powo.science.kew.org/>; accessed on 20 February 2022) and the Germplasm Resources Information Network (GRIN; <https://www.ars-grin.gov/>; accessed on 20 February 2022). These databases were chosen because they maintain detailed profiles of a large number of plant species which are frequently updated, provide traceable data sources, and are widely referred by plant biologists to trace the origin of a species ([Martin et al., 2017](#_ENREF_9); [Turbelin et al., 2017](#_ENREF_16)).

We adopted a consensus approach to determine the origin and invasion status of the species in China. The origin status of the species reported in these five sources (three national checklists and two global databases) were first coded as: -1 for native, 1 for alien and 0 for no information. If the origin status of a species was identified as 'native' (0) or 'alien' (1 to 5) to China in majority of these sources (n=803), it was accepted. However, in case of any contradiction between the sources (e.g., two checklists identified a species as alien whereas one database identified it as native) (n=109), peer-reviewed journal articles, published books, and online databases were consulted to establish the origin status of the species. For literature and book search, the Google Scholar database (<https://scholar.google.com/>; accessed on 21 February 2022) was used. The search terms were optimized following a preliminary search based on a handful of relevant articles retrieved by using the names of the species. The optimized search string consists of a combination of species name and nativity (and its synonyms and related words). The advanced search function of the Google Scholar database was used to search for “species name” with at least one of the words “native” OR “endemic” OR “native region” OR “nativity” OR “origin” present anywhere in the article. The search results were screened until the origin status of the species was discerned confidently. The online databases and repositories were retrieved through a Google search (<http://www.google.com/>; accessed on 21 February 2022) and the databases were considered based on the same criteria (i.e., frequently updated and with traceable data sources). At the end of this step, the origin status of the alien species was categorized as – alien (n=811) and native (n=101). In the second step, the status of the alien species (n=811) in the introduction-naturalization-invasion continuum was determined. If the invasion status of a species was identified as 'alien' or 'naturalized' or 'invasive' in China in at least one of these checklists and was not contradicted by the others, it was accepted (n=614). In case of any contradiction between checklists (n=197), invasion status was determined based on the status reported in the latest (publication year) checklist.

# Supplementary Note 2: Details of statistical analysis

The NRI and NTI values were checked for compliance with the assumption of normality (by using the Shapiro-Wilk test function in R) and homogeneity of variance (Levene test by using the *car* package ([Fox and Weisberg, 2019](#_ENREF_4)) in R) to decide parametric and non-parametric tests.

We first related patterns of phylogenetic relatedness to the data types, species categories and environmental factors. Among the environmental variables, climate is likely to influence large-scale patten of phylogenetic structure ([Weigelt et al., 2015](#_ENREF_17)). Two bioclimatic variables, namely mean annual temperature (BIO1) and annual precipitation (BIO12), were considered in this study. These two bioclimatic variables can drive distributions of plant species and community composition, and therefore, have been included in previous studies on community assembly (e.g., ([Qian and Sandel, 2017](#_ENREF_13)). The raster data of the variables were downloaded from the Worldclim database version 2 ([Fick and Hijmans, 2017](#_ENREF_3)) at 2.5 arcminute resolution (approximately 5 km at the equator). By using ArcMap version 10.2.1, a fishnet of the same cell size to the rasters was created and the values of these bioclimatic variables were extracted for each of the fishnet centroid points. The mean values of the bioclimatic variables were then calculated for each of the four spatial scales (7 MHTs, 16 KGCs, 34 PROs and 47 ECOs). Logistic regression models were fitted (by using the *glm* function in R) with the two data types (presence and abundance), six species categories (introduced, naturalized, invasive, introduced-invasive, introduced-naturalized, naturalized-invasive) and two bioclimatic variables (mean annual temperature and annual precipitation) to check their influences on variation in NRI and NTI values. The categorical variables (data types and species categories) were coded as binary dummy (K-1) variables. The models were developed at each spatial scale, for the three species, and compared based on the second order Akaike’s information criterion (AIC_C_) values (by using the *AICcmodavg* package ([Mazerolle, 2020](#_ENREF_10)) in R).

The NRI and NTI values of the selected models (those with lower AIC_C_ values) were compared considering the species categories and spatial scales by two-way ANOVA. For non-parametric tests, we used the aligned ranks transformation ANOVA (ART anova) by using the *ARTool* package ([Wobbrock et al., 2011](#_ENREF_18)) in R, in which the posthoc pairwise comparisons were done by using the ART-C procedure (with p-values adjusted with the Holm method for multiple comparisons) ([Elkin et al., 2021](#_ENREF_2)). We observed significant differences in NRI and NTI values between the three species categories and four spatial scales; therefore, for a better understanding, NRI and NTI values were further compared between the species categories for each spatial scale separately (parametric or non-parametric test). For parametric tests, we used one-way ANOVA and post-hoc Tukey’s HSD test (using the *multcomp* package ([Hothorn et al., 2008](#_ENREF_5)) in R), whereas for the non-parametric tests, the Kruskal-Wallis H and posthoc Dunn’s test (using the *FSA* package ([Ogle et al., 2022](#_ENREF_11)) in R) were used. The p-values in all tests were adjusted with the Holm method. Finally, Spearman’s rank correlation (the rho coefficient, ρ) was calculated by using the *cor.test* function in R to measure the strength and direction of the relationship between the phylogenetic relatedness among the three species categories at each of the four spatial scales. For p-values with ties, we used the midranks, i.e., by assigning to each tied individual the average of the tied ranks ([Tilquin et al., 2003](#_ENREF_15)). All statistical analyses were conducted both at species and family-levels.

# Supplementary Figure

**
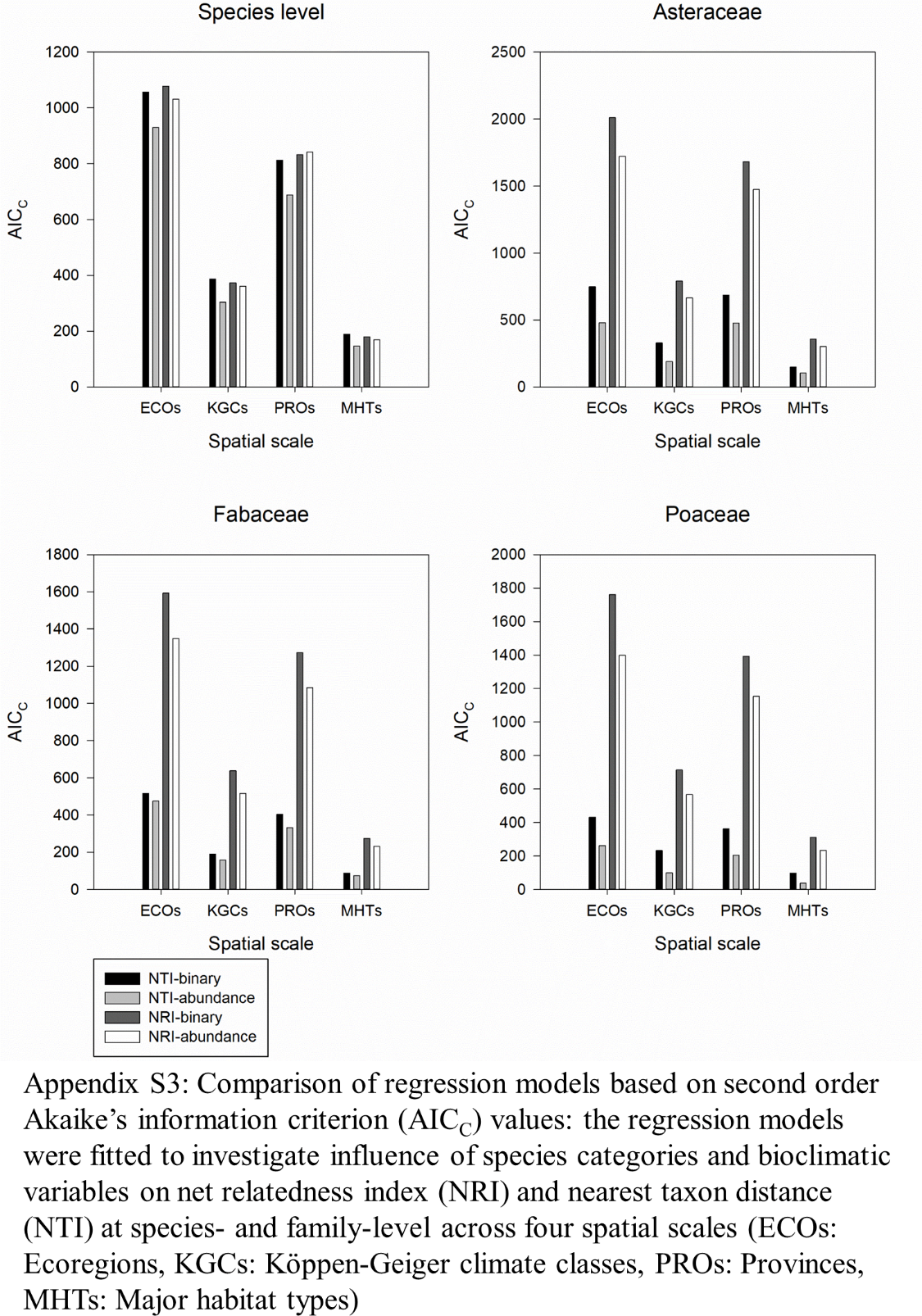
**

**Supplementary Figure 1.** Comparison of regression models based on second order Akaike’s information criterion (AIC_C_) values: the regression models were fitted to investigate influence of species categories and bioclimatic variables on net relatedness index (NRI) and nearest taxon distance (NTI) at species- and family-level across four spatial scales (ECOs: Ecoregions, KGCs: Koppen-Geiger climate classes, PROs: Provinces, MHTs: Major habitat types)

**References**

Borsch, T., Berendsohn, W., Dalcin, E., Delmas, M., Demissew, S., Elliott, A., Fritsch, P., Fuchs, A., Geltman, D., Güner, A., Haevermans, T., Knapp, S., Le Roux, M.M., Loizeau, P.-A., Miller, C., Miller, J., Miller, J.T., Palese, R., Paton, A., Parnell, J., Pendry, C., Qin, H.-N., Sosa, V., Sosef, M., Von Raab-Straube, E., Ranwashe, F., Raz, L., Salimov, R., Smets, E., Thiers, B., Thomas, W., Tulig, M., Ulate, W., Ung, V., Watson, M., Jackson, P.W., and Zamora, N. (2020). World Flora Online: Placing taxonomists at the heart of a definitive and comprehensive global resource on the world's plants. *TAXON* 69**,** 1311-1341.

Elkin, L.A., Kay, M., Higgins, J.J., and Wobbrock, J.O. (Year). "An aligned rank transform procedure for multifactor contrast tests", in: *Proceedings of the ACM Symposium on User Interface Software and Technology (UIST '21)*: ACM Press), 754-768.

Fick, S.E., and Hijmans, R.J. (2017). WorldClim 2: new 1-km spatial resolution climate surfaces for global land areas. *International Journal of Climatology* 37**,** 4302-4315.

Fox, J., and Weisberg, S. (2019). *An R Companion to Applied Regression.* Thousand Oaks CA: Sage.

Hothorn, T., Bretz, F., and Westfall, P. (2008). Simultaneous inference in general parametric models. *Biom J* 50**,** 346-363.

Jiang, H., Fan, Q., Li, J.-T., Shi, S., Li, S.-P., Liao, W.-B., and Shu, W.-S. (2011). Naturalization of alien plants in China. *Biodiversity and Conservation* 20**,** 1545-1556.

Kindt, R. (2020). WorldFlora: An R package for exact and fuzzy matching of plant names against the World Flora Online taxonomic backbone data. *Applications in Plant Sciences* 8**,** e11388.

Ma, J., and Li, H. (2018). *The Checklist of the Alien Invasive Plants in China.* Beijing: Higher Education Press.

Martin, P.A., Newton, A.C., and Bullock, J.M. (2017). Impacts of invasive plants on carbon pools depend on both species’ traits and local climate. *Ecology* 98**,** 1026-1035.

Mazerolle, M.J. (2020). "AICcmodavg: Model selection and multimodel inference based on (Q)AIC(c). R package version 2.3-1, https://cran.r-project.org/package=AICcmodavg".).

Ogle, D.H., Doll, J.C., Wheeler, P., and Dinno, A. (2022). "FSA: Fisheries Stock Analysis. R package version 0.9.3, https://github.com/fishR-Core-Team/FSA".).

Pyšek, P., Richardson, D.M., Rejmánek, M., Webster, G.L., Williamson, M., and Kirschner, J. (2004). Alien Plants in Checklists and Floras: Towards Better Communication Between Taxonomists and Ecologists. *Taxon* 53**,** 131-143.

Qian, H., and Sandel, B. (2017). Phylogenetic relatedness of native and exotic plants along climate gradients in California, USA. *Diversity and Distributions* 23**,** 1323-1333.

R Core Team (2020). "R: A Language and Environment for Statistical Computing". (Vienna, Austria: R Foundation for Statistical Computing).

Tilquin, P., Van Keilegom, I., Coppieters, W., Le Boulengé, E., and Baret, P.V. (2003). Non-parametric interval mapping in half-sib designs: use of midranks to account for ties. *Genetical Research* 81**,** 221-228.

Turbelin, A.J., Malamud, B.D., and Francis, R.A. (2017). Mapping the global state of invasive alien species: patterns of invasion and policy responses. *Global Ecology and Biogeography* 26**,** 78-92.

Weigelt, P., Daniel Kissling, W., Kisel, Y., Fritz, S.A., Karger, D.N., Kessler, M., Lehtonen, S., Svenning, J.-C., and Kreft, H. (2015). Global patterns and drivers of phylogenetic structure in island floras. *Scientific Reports* 5**,** 12213.

Wobbrock, J.O., Findlater, L., Gergle, D., and Higgins, J.J. (Year). "The aligned rank transform for nonparametric factorial analyses using only ANOVA procedures", in: *Proceedings of the ACM Conference on Human Factors in Computing Systems (CHI '11)*: ACM Press, New York), 143-146.

Zhao, C., Liu, Q., Li, F., Wong, L.J., and Pagad, S. (2020). Global Register of Introduced and Invasive Species - China. Version 1.3. Invasive Species Specialist Group ISSG. Checklist dataset. *Accessed via GBIF.org on 2022-02-20 https://doi.org/10.15468/wstyjh*
